# A balanced view of impossible aesthetics: An empirical investigation of how impossibility relates to our enjoyment of magic tricks

**DOI:** 10.1177/20416695221142537

**Published:** 2023-01-02

**Authors:** Steven E Bagienski, Gustav Kuhn

**Affiliations:** 4898 Goldsmiths University of London, London, UK

**Keywords:** magic, aesthetics, emotions, enjoyment, impossibility

## Abstract

The performance art of magic allows us to experience the impossible, and this study used a balancing magic trick to investigate the relationship between participants’ enjoyment and perceived impossibility. Participants watched a live performance of a magic trick in which the magician balanced objects in progressively more impossible configurations. At seven different time points observers rated their enjoyment, and the extent to which they believed what they saw was impossible. Regression analysis revealed that participants’ enjoyment of the magical effect relates to their perceived impossibility of the magic trick, and this relationship was independent of how much they enjoyed magic in general. Moreover, a one-way within-subjects analysis of variance showed that participants enjoyed the performance More as the trick became more impossible. However, once the magical effect was anticipated, enjoyment began to plateau while perceived impossibility continued to increase. These results are discussed in the context of people's aesthetic appreciation of magic and current arts appreciation models.

Stage magic allows us to experience the impossible, and this sense of impossibility lies at the core of this performance art. Magic is an artform^1^ that is enjoyed across the globe, and these illusions have captivated people for many centuries. In recent years there has been much interest in understanding the psychological mechanisms that underpin the creation of these illusions (Kuhn et al., 2008, 2016; Macknik et al., 2008; Rensink & Kuhn, 2015; Thomas et al., 2015), and this theoretical work has activated a whole research community that has empirically investigated the cognitive mechanisms of such illusions (e.g., Ekroll et al., 2018; Ekroll et al., 2016; Ekroll et al., 2013; Hergovich et al., 2011; Kuhn & Land, 2006). However, there is very little empirical work examining why people enjoy these impossible moments. In fact, despite the popular appeal of such magical illusions, there are only a handful of theoretical frameworks to explain why or how people enjoy magic (Leddington, 2016a, 2017; Grassi & Bartels, 2021; Kuhn, 2019). Magic is unique in that it allows us to experience things that we believe to be impossible (Kuhn, 2019), and in this paper we examine the link between enjoyment and the perceived impossibility that such illusions elicit.

There has been much theoretical and empirical work that examines our enjoyment of different artforms (e.g., Bullot & Reber, 2013; Rose et al., 2020; Ruth & Müllensiefen, 2020). For example, Bullot and Reber (2013) proposed an influential model of arts appreciation, which discusses three modes of appreciation: basic exposure, the artistic design stance, and artistic understanding. The artistic design stance is “an attitude whereby appreciators develop their sensitivity to art-historical contexts by means of inquiries into the making, authorship, and functions of artworks” (Bullot & Reber, 2013, p. 123). For example, people appreciate a painting more when given descriptions about the artist's technique, the materials used, and other information about the artist's style (Belke et al., 2006). It is hard to see how this concept can be directly implemented to explain people's enjoyment of magic. Magic is unique in that the performer intentionally withholds information about the method and material used to create the performance, and it is this secrecy and deception that enables the experience of the impossible. Unlike most other artforms, the true authorship of magical effects is also frequently absent to prevent the audience from discovering the secrets. Most other arts allow us to marvel at the creation of the artwork (e.g., the artist's effort and skills). However, people appreciate magic *because* this aspect is withheld, in the aim of creating an impossible experience. While an appreciation of the art-historical context can still exist (e.g., magicians debunking paranormal claims during a spiritualism movement), the majority of the artistic design stance is absent. This psycho-historical framework is therefore limited in that it does not distinguish how arts can be enjoyed when the artistic design stance is largely absent. Thus, we must look elsewhere for insights on why we enjoy magic.

The most comprehensive theories on why we enjoy magic originate from philosophy and the work of magicians. Leddington suggests that the experience of magic results from a cognitive conflict, which arises from an incongruity between perceiving something as impossible and knowing that our perception is fake, yet lacking any evidence that our perception is fake (Kuhn, 2019; Lamont, 2013; Leddington, 2016a). As a result, this can yield a heightened sense of curiosity as the magician provides ample counterevidence for any rational explanation that a spectator can surmise (Leddington, 2016b). This heightened curiosity is one emotion that may play a role in our enjoyment of magic.

It is important to note that magic can elicit a range of pleasant, unpleasant, or mixed emotions, such as awe and surprise, both of which can have positive or negative valance (Bagienski & Kuhn, 2019; Lesaffre et al., 2020; Noordewier & Breugelmans, 2013; Yaden et al., 2018). One explanation for how we can enjoy this complex set of emotions stems from the distancing-embracing model, which suggests that the safe context in which magic is performed allows the viewer to distance themselves from the negative emotion. This distancing process allows the viewer to embrace the negative emotions and consequently to reappraise them in a more favorable light. This distancing and embracing mechanism allows us to enjoy the overall experience (Leddington, 2017; Menninghaus et al., 2017). Elaborating on this, Leddington takes it one step further by arguing that watching a magic performance elicits an oscillation between the emotions of confusion and interest (Leddington, 2019, 2021).

This oscillation between emotions may relate to the concept of Semantic Instability which has become an influential psycho-aestetic concept to help explain our interest and enjoyment semantically instable objects such as when objects can be interpreted in conflicting ways (Muth & Carbon, 2016; Muth et al., 2018). Muth and Carbon suggested that the process of disambiguating semantically undefined data can be rewarding, and that even the anticipation of such insights may account for its aesthetic appeal. In the context of a magic show there can be an oscillation of having nearly solved how the trick is done, followed by having that solution quashed and thus no longer understanding how the effect has been achieved (Tamariz, 1988). A successful magic performance will never resolve the secret method to the trick, but this oscillation between knowing and not knowing may account for some of our interested and enjoyment of magic.

Further insights into why we enjoy magic come from experienced magicians, which resemble some of these ideas put forth by Leddington (2016a). For example, Teller of *Penn and Teller* describes magic as “a form of theatre that depicts impossible events as though they were really happening” (Stromberg, 2012). Similarly, Darwin Ortiz has written extensively on magic theory, and he states that magic is about creating “an illusion, the illusion of impossibility” (Ortiz, 2006, p. 30). The imaginative magic creator, Simon Aronson, describes the magic experience as

[the spectator] knows what has just happened, and yet also knows that it cannot happen, that it defies the controlling laws that govern our world. And yet, you did it. A magician's paramount goal is to manipulate the spectator's mind and senses to bring about this state of impossibility. (Jay et al., 2013, p. 35)

Or as Leddington states it, “the distinctive aim of theatrical magic is to produce an experience as of an impossible event” and the value of understanding the magic experience is in its uniqueness from other arts as “a first step toward a general aesthetics of the impossible” (Leddington, 2016a, p. 254).

To date, very little empirical research has directly investigated the emotions that magic elicits or the psychological factors that modulate our enjoyment of magic. At the center of this experience lies experiencing things that appear to be impossible. Neurological studies support this view and demonstrate that magic tricks elicit neural activations in brain areas that are involved in experiencing and resolving cognitive conflicts (Danek et al., 2015; Parris et al., 2009). Griffiths (2015) has shown that different types of magical transformations elicit more or less interest in the magical effect, which suggests that our enjoyment of magic is directly related to internal world views. Danek et al. (2014) has also used magic tricks to investigate insight experiences, and found that discovering secrets to tricks were most associated with a sense of happiness with qualitative data revealing themes of pride and tension release. Curiosity arousing stimuli were also developed using ratings of surprise, interest, clarity of trick, and confidence in solution (Ozono et al., 2021) and found positive correlations amongst surprise, curiosity, and interest whereas confidence in solution (i.e., impossibility) was negatively correlated with the rest. These studies all point to magic eliciting emotions that promote knowledge exploration like surprise, curiosity, and confusion (Vogl et al., 2020), which arise from an unexplainable moment of the impossible.

Taken altogether, an empirical investigation of magic enjoyment, first and foremost, ought to begin by examining its relationship with the perceived impossibility of magic performances. We set out to do so by utilizing a magic trick that produces different degrees of “impossibility” within the same performance. We avoided magic tricks where the magic moment was a vanish, appearance, or transformation because these magic moments often occur quickly, without a gradual increase in impossibility. Furthermore, such effects make it difficult to define a baseline where the magician performs a similar action that appears possible (see Parris, et al., 2009). We therefore settled on a trick that involves balancing objects in ways that are possible, pseudo-possible, and impossible (Jay, 2018). This allows a very plausible baseline to be established, while the performance grows progressively more impossible until it borders the resemblance of a completely impossible levitation. Furthermore, since humans have systematic balance estimation biases (Firestone & Keil, 2016; Øhrn et al., 2019), a balancing trick will likely provide a better variance/middle ground in spectators’ possibility ratings to avoid extreme scores.

In the current work, we used this balancing trick to investigate the relationship between participants’ enjoyment and perceived impossibility. We hypothesized that viewers perceive the performance to be progressively more impossible as time went on, and that impossibility would be strongly correlated with enjoyment. That is, more impossible moments would be more enjoyable and vice versa.

## Method

### Participants

Participants were 185 first-year psychology undergraduate students attending a research methods lecture, where they were invited to take part in ongoing research studies within the psychology department (data from 56 participants were excluded due to incomplete questionnaire completion). The average age was 20.0 (SD = 4.19) consisting of 106 women and 23 men. Students were rewarded with research participation credits that counted toward their final grade in the course. The Psychology Department provided ethical approval for the two experiments. All variables that have been collected are reported.

### Procedure and Magic Performance

The magic trick was performed live at the front of a lecture hall for students who were directed to the online questionnaire before the performance began. The trick, *Balance*, was created by Joshua Jay (Jay, 2017, 2018) and involved the magician stacking numerous items to form an impossible balancing act. The trick used a bottle, a toothbrush, a pencil, an empty card box, an empty crayon box, and a blue crayon. All objects were first handed out to a few students for examination before the trick began. The performer maintained a neutral emotional expression throughout the performance and the script was limited to instructions on when to proceed to the next pages of the questionnaire. These instructions corresponded to the seven timepoints of the magic performance that we measured their enjoyment and the perceived impossibility.

**Figure 1.**
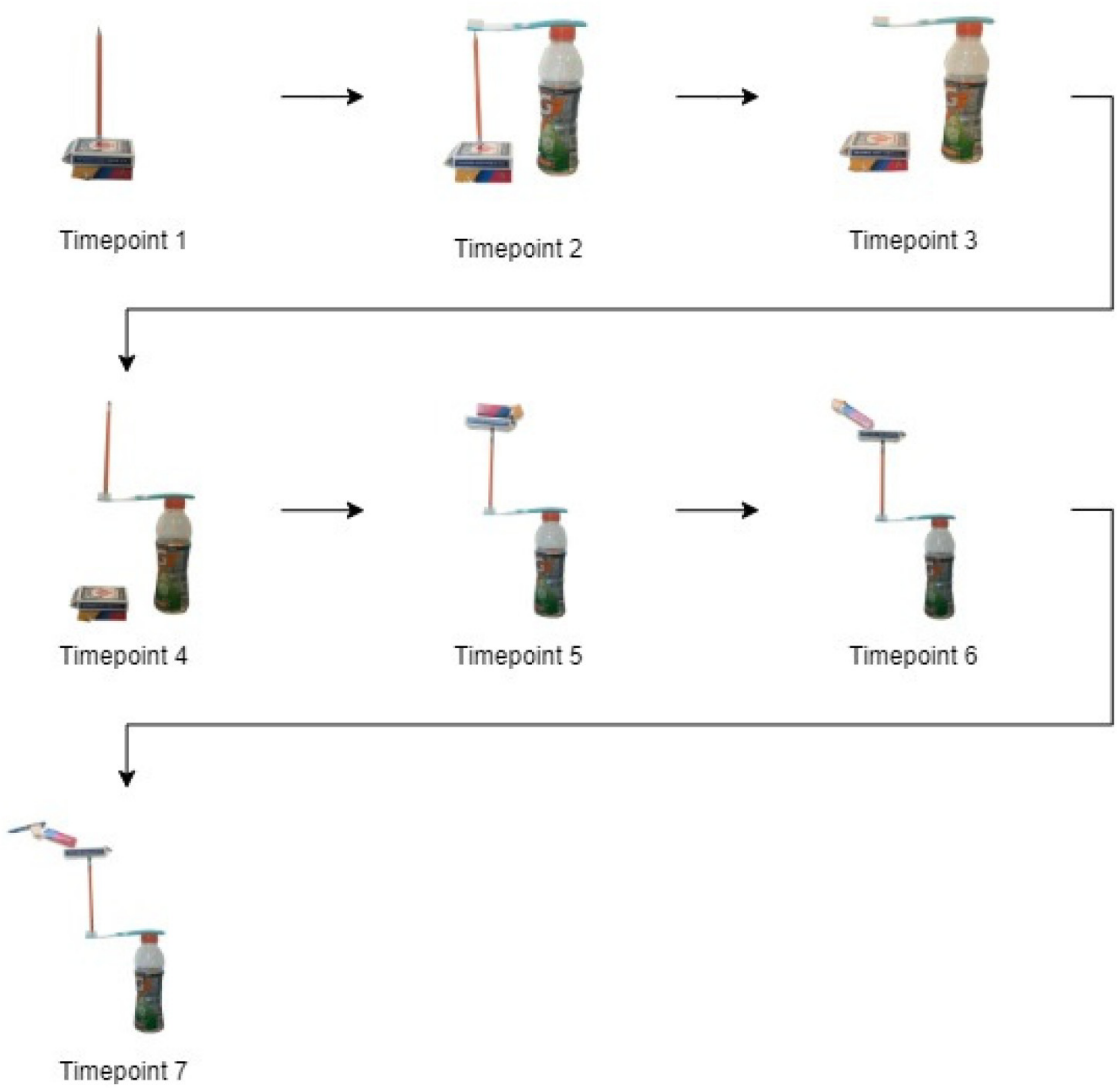
The seven timepoints of the balancing magic trick where impossibility and enjoyment measures were taken. Each timepoint reflects a stable, self-standing structure.

The performance began by balancing the objects in a plausible manner, such as balancing the pencil on its flat eraser end or creating a bridge from the objects. The balancing became progressively more impossible during each timepoint. The full progression of the impossible balancing act is outlined in Figure 1. After the final timepoint rating, participants were asked to give an overall rating for how impossible and enjoyable the magic performance was.

### Measures of Enjoyment and Perceived Impossibility

The questionnaire was administered via Qualtrics online software. The students completed the questionnaire on their laptop or device of their choice. All items utilized a continuous slider scale with values ranging from 0 to 100.

Before the performance, participants answered the question “How much do you enjoy watching magic tricks (i.e., tricks performed for entertainment)?,” with scale labels ranging from *I do not enjoy watching magic tricks at all* to *I enjoy watching magic tricks more than anything else*. This was used to ensure that their enjoyment of *Balance* was not a bias of their general magic enjoyment, which was analyzed by examining the extent to which people's general enjoyment predicts their enjoyment of *Balance*.

After observing each timepoint of the magic performance, participants answered two questions. To assess enjoyment, the questionnaire asked, “How much did you *enjoy* what you just saw?,” with scale labels ranging from “I did not enjoy it at all” to “I enjoyed it immensely.” To assess perceived impossibility, students were asked “How *impossible* was the thing you just saw?,” with scale labels ranging from “Completely possible” to “Completely impossible.” At the end of the performance, students answered “Overall, how much did you enjoy the entire demonstration?,” and “Overall, how impossible was the entire demonstration?” with the same scale labels as the prior questions.

## Results

### Regression of Overall Enjoyment, Overall Impossibility, and General Enjoyment

To control for potential bias and confounds from participants general enjoyment of magic, our first analysis was a regression analysis predicting the actual enjoyment of our live magic performance using the overall impossibility of *Balance* and general magic enjoyment as predictors. All variables were assessed for normality via the values for skewness (absolute value less than 2) and kurtosis values (absolute value less than 7) as per Curran et al. (1996). The data for the regression did not meet the assumption of homoscedasticity, so we used the macro developed by Hayes and Cai (2007) for adjusted standard errors. Tolerance levels indicated no multi-collinearity. The regression indicated that the model was a significant predictor of overall *Balance* enjoyment, model *R*^2^ = .309, *F*(2, 125) = 27.4, *p* < .0001). General enjoyment of magic was a significant predictor of our trick's enjoyment (β = .424, *t* = 3.91, *p* < .0001) and overall impossibility of our trick was also significant (β = .327, *t* = 4.00, *p* < .0001).

### Enjoyment and Impossibility Correlation Across Time

**Figure 2.**
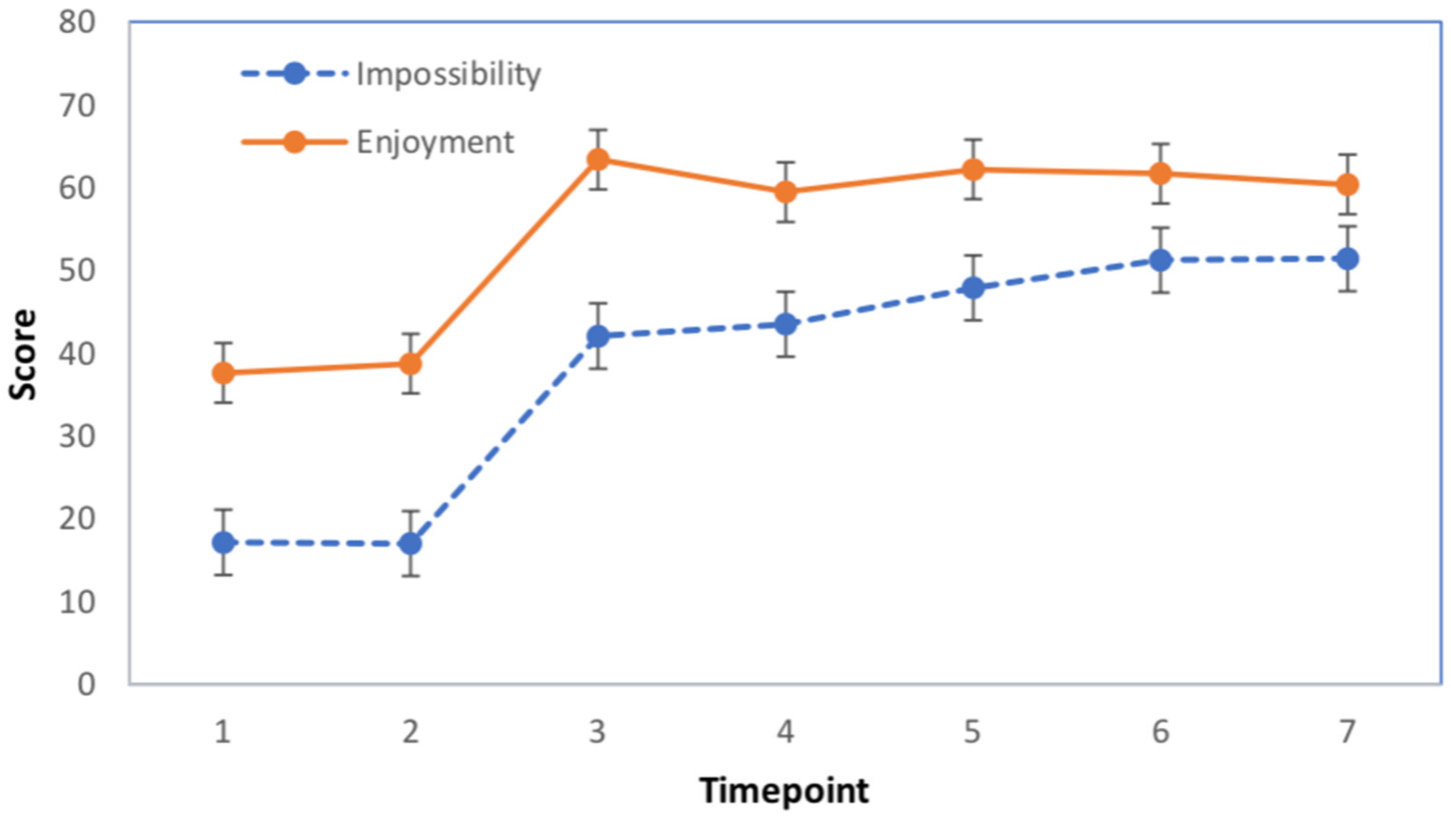
Mean impossibility and enjoyment scores across all seven timepoints of the magic performance. Timepoints 1 and 2 reflect the only timepoints where the balancing was reasonably possible.

The next analysis examined the relationship between perceived impossibility and enjoyment of the trick across the seven time points (see Figure 2). Variables were assessed for normality via the values for skewness and kurtosis in the same manner as the regression. Participants were excluded from the data analysis if they failed at least one attention check, did not complete the questionnaire, or if the participant gave the same rating for all timepoints (i.e., undefined correlation coefficient). This resulted in 123 participants (102 female, 21 male) in the final sample for analysis with a mean age of 20.0 (SD = 4.19).

To analyze the progression of enjoyment and impossibility across time, two one-way within-subjects analysis of variance were conducted with a Huynh-Feldt correction. Enjoyment scores differed statistically significantly across timepoints, *F*(3.45, 442) = 68.3, *p* < .001, partial η^2^ = 0.348. Bonferroni corrected pairwise comparisons confirmed that both the first and second timepoints were significantly less enjoyable than all subsequent timepoints where the impossible moments had occurred (see Figure 2). After the first impossible moment occurred, enjoyment plateaued with no additional statistically significant results in post-hoc analyses.

Similar to enjoyment, impossibility ratings differed significantly across timepoints, *F*(3.26, 417) = 106, *p* < .001, partial η^2^ = 0.455. Bonferroni corrected pairwise comparisons again confirmed that both the first and second timepoints were significantly less enjoyable than all subsequent timepoints. Unlike enjoyment, however, impossibility increased gradually as the performance continued, with both the first and second impossible moments (i.e., timepoints 3 and 4) scoring lower on impossibility than the two final timepoints (see Figure 2). Similarly, impossible timepoint 3 was significantly lower than timepoint 5, whereas neither of these timepoints were statistically different than timepoint 4. Numerical values of the means also indicated a gradual increase of impossibility.

Next, we examined the relationship between impossibility ratings and enjoyment over time. Spearman's correlation coefficients were first calculated for each participant by using their scores at all seven timepoints. A one sample *t*-test showed that the mean correlation coefficients were significantly greater than zero (*M* = 0.564, SD = 0.393, 95% CI [0.493, 0.634]), *t(*122) = 15.83, *p* < .001, Cohen's *d* = 1.43. These results further illustrate a strong positive relationship between perceived impossibility and enjoyment.

## Discussion

This study aimed to investigate the relationship between people's enjoyment and their perceived impossibility of magic. Our regression analysis shows that people's enjoyment of the magical effect relates to their perceived impossibility of the magic trick, and this relationship was independent of how much they enjoyed magic in general. Moreover, our correlational analysis showed that participants enjoyed the performance more as it became more impossible. However, this correlation was not perfect. After the first impossible moment had occurred, enjoyment plateaued whereas perceived impossibility continued to increase as additional objects were added to the impossibly balanced structure. At first, it may appear perplexing why enjoyment levels off if perceived impossibility continues to increase. However, we believe that this occurs because most of the enjoyment arises from the *unexpected surprise* of witnessing an impossible moment. In our case, enjoyment was predominantly derived from the *first* impossible balancing moment. Once these impossible balancing acts were anticipated, subsequent repetitions with other objects are less surprising and enjoyment does not increase further, despite the structure becoming more impossible. Indeed, a previous questionnaire-based study showed that people particularly appreciate the element of surprise that magic offers (Jay, 2016). Moreover, preliminary data from our own lab shows that people rate surprise as most important emotion when asked to identify the emotions that magic elicits. Our study is unique in that it allows us to examine participants’ enjoyment of the trick as the performance unfolds. It is likely that spectators begin to rationalize how the first impossible moment occurred. As time progresses, they have more time to rationalize and as they persistently fail to find an adequate explanation, their impossibility evaluation increases. This effect is strengthened by adding more impossibly balanced objects, which occurs at a faster rate than the spectator can generate adequate explanations for what had occurred. This results in a cognitive overload and ultimately higher impossibility ratings. It is also worth noting that at timepoint 4 the magician stabs a pencil into the bristles of the toothbrush. This balancing act seems plausible, which may explain why we perceive no increase in impossibility between timepoint 3 and timepoint 4.

Not being able to figure something out should become frustrating, but magicians frame their performance by keeping the expectation that you can “solve” it by just looking more closely. This expected solvability can sustain curiosity and keeps frustration at bay. Accordingly, we can enjoy uncertainty/incongruity (Van der Cruys et al. 2022) as long as there is a metacognitive expectation that one can deal with it, despite this in reality not being the case. Even though the spectators may feel cheated after once the trick has been completed, more positive emotions such as awe (for dexterity, showmanship, cleverness) now start to dominate.

Magic is an artform that allows us to experience the impossible, and our results illustrate that people enjoy experiencing this sense of impossibility. These results dovetail some of the theoretical works on magic which describe the experience of magic as an intellectual rather than emotional experience (Leddington, 2016a). Kuhn (2019) has suggested that our enjoyment of magic results from an inherent drive towards exploring things that violate our understanding of the world. Infants are captivated by events that violate their understanding of the world, and it has been suggested that our attraction and enjoyment of magic may result from this captivation of the impossible (Harris, 1994). Lewry et al. (2021) have reported a negative correlation between adults’ interest in different types of magical transformation and the age in infancy at which they learned that the transformations violated their understanding of the world. These results point towards a link between interest in magic and the strength of our beliefs that such transformations are impossible. Moreover, Medeirose et al., (2021) have recently examined some of the attributes that people particularly enjoy about magic. To do so, they asked people to describe the things that they enjoyed about magic. One of the most common themes to emerge from this analysis was violations in the laws of nature—in other words experiencing things that appear impossible.

We have conducted a related experiment which adds further weight to the relationship between impossibility and enjoyment. In this study (Kuhn, et al., submitted), we examined amongst other things, the impact that both objective and subjective impossibility have on people's enjoyment of magic. Participants watched a magic trick in which the spectator was invited to think of a number, which the magician magically managed to devine. There were different versions of this trick, which were all identical, with the exception of the number range from which the spectator was allowed to select their number (1-4, 1-10, 1-100, 1-1,000, 1-100,000, any). In doing so the experimenters manipulated the objective probability by which the magic trick could have been achieved by chance. Their results showed that this objective probability had no direct impact on participants’ enjoyment of the trick, but there was a strong correlation between the extent to which they rated the trick to have been impossible and their enjoyment of the trick. These results add further weight to the argument that our enjoyment of magic directly results from experiencing things we believe to be impossible.

Magic is an artform that encompasses a wide range of genres, and they all vary in terms of the types of effects that are performed (e.g., stage illusions, close up, mentalism…) as well as the way they are performed (e.g., comedy, bizarre, spectacle, dance, etc.). We chose a magic trick that was devoid of any specific performance genre, in the hope of capturing the essence that appears central to all magic—experiencing the impossible. That said, there are obvious limitations in focusing on one single piece of magic, as this limits the extent to which our results generalize to other performances. Most past research that has examined the emotions that magic elicits by asking participants to watch a range of different magic tricks (Danek et al., 2014, 2015; Ozono et al., 2021; Parris et al., 2009). There is an inherent advantage in doing so, but in each of these studies, the tricks were performed by a limited number of performers, and they were all performed in the same style. Further research may try to examine whether our findings generalize to other magic performances and illusions.

Another limitation might be how participants interpret the word “enjoyment,” since magic operates more on the intellectual side (Leddington, 2016a) and thus, engagement may be more akin to an intellectually challenging flow-like experience, whereas “enjoyment” may have been interpreted more like a joyous happiness. To address the broad interpretations of “enjoyment,” one approach would be for future studies to investigate a more comprehensive list of emotions to better understand the emotional fingerprint of the magic experience. This would help clarify which emotions are most important to the experience. Considering the visual nature of magic tricks, the 27 emotions elicited by videos in Cowen and Keltner's research (2017) would be appropriate. Some of these emotions might be surprise, confusion, curiosity, and wonder. In psychology, wonder is typically used interchangeably with awe, and like magic, one feature of awe is a need for cognitive closure (Keltner & Haidt, 2003; Yaden et al., 2018). Thus, measuring awe in more detail during the magic experience would likely provide deeper insights into the magic experience. Similarly, curiosity would be worth investigating further because while an experience of awe is independent of whether the cognitive conflict is resolved, feeling curious (or confused) implies that the conflict remains unresolved, as is the case in magic.

Future work aimed at understanding or improving magic performances can focus on several different areas. Research on dialectical thinking (e.g., Hui et al., 2009; Kitayama et al., 2010; Miyamoto & Ryff, 2011; Shiota et al., 2010), or the tendency to accept contradictory thoughts and emotions, would be relevant to the enjoyment of negative emotions in magic (Leddington, 2017) with individual differences yielding valuable insights into how audiences enjoy magic. Further exploration into the role of *unexpected* surprise may also prove fruitful. For example, future experiments could investigate how enjoyment is affected by the number of unexpected impossible moments within a performance or by whether or not an impossible moment was anticipated. A vast number of such moments within the same performance may be especially relevant to Awe since vastness has been a common theme in research on awe (Keltner & Haidt, 2003; Yaden et al., 2018).

In summary, the present study was the first to empirically show a relationship between impossibility and enjoyment of a magic performance, where higher perceived impossibility correlated with enjoyment. However, it appears that once the magical effect is anticipated, enjoyment begins to plateau while perceived impossibility can continue to increase. Therefore, we believe surprise of an unexpected, impossible moment to be driving the enjoyment in magic.

## Notes

1. There is some debate as to whether magic is considered an artform or not, the performance art of magic, and magic is often not recognized within more traditional arts organisations. However, in 2016 the US Congress passed a bill stating that magic is a rare and valuable art form (H.Res.642, 2016). For a more detailed discussion, see Steinkraus (1979).
